# Comparison of joint awareness after total knee arthroplasty, medial unicompartmental knee arthroplasty, and high tibial osteotomy: a retrospective study

**DOI:** 10.1186/s12891-023-06779-y

**Published:** 2023-08-24

**Authors:** Yiwei Huang, Hao Ge, Bo Peng, Wenjun Feng, Haitao Zhang, Yirong Zeng

**Affiliations:** 1 The First Clinical of Medical School, Guangzhou University of Chinese Medicine, NO.12 Jichang Road, District Baiyun, Guangzhou, 510405 Guangdong China; 2 Department of Orthopaedics, The First Affiliated Hospital of Guangzhou University of Chinese Medicine, NO.16 Jichang Road, District Baiyun, Guangzhou, 510405 Guangdong China; 3 Longhua Hospital, Shanghai University of Traditional Chinese Medicine, No. 725, Wanping South Road, Shanghai, 200032 China

**Keywords:** Forgotten Joint Score-12(FJS), High tibial osteotomy (HTO), Unicompartmental knee arthroplasty (UKA), Total knee arthroplasty (TKA)

## Abstract

**Introduction:**

This study aimed to compare the Forgotten Joint Score-12(FJS) outcomes and the minimum clinically important difference (MCID) of the FJS after high tibial osteotomy (HTO), unicompartmental knee arthroplasty (UKA), and total knee arthroplasty (TKA) with short-term follow-up (at least 2 years). Another objective of the study is to investigate the factors influencing FJS. It is hypothesized that there are differences in FJS outcomes among the three procedures.

**Methods:**

Patients who underwent HTO, UKA, and TKA from January 2016 to December 2020 and were followed up for a minimum of 2 years were included in the study. The FJS were analyses from a cohort of people who submitted data to two years. The preoperative and postoperative clinical outcomes were compared and evaluated the patient-related factor. The FJS scores were predicted using multiple linear regression analysis. Additionally, Patient's Joint Perception (PJP) questions were used as anchors to determine the achievement of the forgotten joint, and FJS MCID were calculated using the receiver operating characteristic curve (ROC).

**Results:**

Three hundred eighty-nine patients were included in the final study, and there were 111 patients in HTO groups,128patients in UKA groups, and 150 patients in TKA groups. The mean follow-up was 47.0 months. There was a significant difference in the total FJS, between the HTO, UKA, and TKA groups (FJS:59.38 ± 7.25, 66.69 ± 7.44 and 56.90 ± 6.85, *p* < 0.001. We found the MCID of the FJS of HTO, UKA, and TKA were 63.54, 69.79, and 61.45, respectively. In multiple linear regression, younger age, and higher FS were significant predictors of better FJS.

**Conclusion:**

Medial UKA demonstrated lower patient awareness in comparison to HTO and TKA, as assessed by the FJS. Younger age and higher FS were identified as significant predictors of improved FJS, providing valuable guidance for surgical decision-making.

## Introduction

The incidence of knee osteoarthritis (KOA) is increasing with the rise of the aging population, especially in women, and the lifetime risk of symptomatic knee OA is 50% [1]. KOA manifests several symptoms, such as pain, stiffness, dysfunction, and even deformity, which Reduces the quality of life [2]. Medial KOA affects up to 50% of KOA patients and exhibits lesser changes compared to the lateral and patellofemoral compartments [3].

At present, the mainstream surgical approach for the treatment of medial OA includes total knee arthroplasty(TKA), unicompartmental knee arthroplasty (UKA), and high tibial osteotomy(HTO) [4]. UKA and HTO generally have better postoperative functional outcomes than TKA [5–8]. Although the survival rate in TKA has improved, a relevant number of patients remain unsatisfied with the outcome, and range from 20 to 30%, regardless of specific TKA design features [9, 10]. For medial OA patients, medial compartment UKA is an option for some people with medial compartment KOA and is viable alternative to HTO or TKA [4, 11, 12]. However, HTO appears to be a better choice for younger patients with high functional activity needs [13].

There has some been debate among surgeons regarding procedures choice between the TKA and UKA and HTO since the three procedures share some similar indications. A related study revealed that the propensity for HTO to be used in more active patients and UKA to be performed in patients who are preoperatively more sedentary [14]. A previous meta-analysis showed that revision to TKA after UKA occurred 8.2 years after surgery, whereas revision to TKA after HTO occurred 9.7 years after surgery [15].

However, the predominance of the three procedures is inconclusive. Maybe the outcome measures used in previous studies are inadequate to distinguish three procedures. One potential explanation for this phenomenon is the significant conceptual differences among TKA, UKA, and HTO. The observed variance may be attributed to the resection of joint surfaces in TKA and UKA, preservation of joint surfaces in HTO, and substantial changes in knee alignment in HTO and TKA compared to minimal changes in UKA [16, 17]. These different procedures can lead to variations in patient awareness after surgery, which can be assessed using the Forgotten Joint Score-12 (FJS) [17]. In general, healthy joints do not exhibit joint awareness, while unhealthy joints often manifest symptoms such as pain, instability, and functional limitations [18]. FJS is composed of 12 items to detect patients’ awareness of their knees in daily life [19]. The utilization of FJS scores for assessing joint awareness exhibited a limited ceiling effect and may provide helpful information for surgeons counseling patients considering HTO、UKA or TKA [18].

The primary objective of this study is to compare the outcomes of the FJS and determine the FJS MCID among patients TKA, UKA, and HTO. Another objective is to examine the factors that influence the FJS. Additionally, we aim to investigate whether UKA yields superior FJS outcomes and lower joint awareness. It is worth noting that, to the best of our knowledge, this is the first study to assess and compare FJS outcomes and define MCID for a forgotten joint in a population of patients undergoing HTO, UKA, and TKA.

## Materials and methods

### Patients and data collection

This retrospective, single-center study collected medical records anonymously and retrospectively from January 2016 to December 2020. Ethical approval for data collection was granted by the Ethics Committee and complied with the Declaration of Helsinki (Research Ethics Approval Code: IIT2022-27). Informed consent was obtained from all participants through clinic-based or telephone communication. The collected baseline and clinical data included age, sex, body mass index (BMI), preoperative Kellgren-Lawrence (KL) grade, American Society of Anesthesiology (ASA), and range of motion. The study recruited 467 patients with KOA scheduled to undergo TKA, UKA, and HTO from January 2016 to December 2020. The three groups were followed for at least two years.

Both UKA and HTO were selectively performed in patients diagnosed with an isolated medial compartment lesion with preserved status in other compartments, and intact anterior and posterior cruciate ligaments [8]. Exclusion criteria [20, 21] were applied, including patients with either lateral or patellofemoral compartmental OA (K-L grade ≥ III), those with knee flexion under 120°, flexion contracture over 20°, or combined ligamental instability, and those with inflammatory arthropathy. Patients who underwent bilateral HTO were also excluded to eliminate confounding factors. Moreover, the operating surgeon intraoperatively assessed the status of the lateral compartment and decided to perform either UKA or TKA. TKA was performed on all patients who did not qualify for UKA and HTO, and on those with apparent lateral and patellofemoral arthritis on preoperative X-rays and grades II-IV on the Kellgren-Lawrence scale [22]. All procedures were carried out by a surgeon with more than 15 years of experience.

### Patient-reported outcome measurements

Patient-reported outcome measures (PROMs) were electronically evaluated using touch-screen devices or mobile phones, beginning in January 2020. These measures included the Forgotten Joint Score-12 (FJS), Knee Society Knee Score (KS), and Function Score (FS). The FJS is a 12-item questionnaire where patients rate their knee function on a 5-point Likert scale: "Never," "Almost never," "Seldom," "Sometimes," or "Mostly" [23]. The Knee Society Knee Score (KS) and Function Score (FS) assess the range of motion, stability, and pain of the knee after surgery [24]. Both scores range from 0 to 100, with higher scores indicating better outcomes.

### Anchor questions for the forgotten joint

In this study, the Patient's Joint Perception (PJP) question served as the anchor question for measuring patients' perceptions of their joint [25]. The PJP question asked patients to rate their joint perception on a scale of 1 to 5, with 1 being "like a native or natural joint" and 5 being "like a nonfunctional joint." This rating system was found to be reliable and valid in identifying the forgotten joint phenomenon [26]. Patients who responded with a rating of "like a native or natural joint" were considered to have a forgotten joint [27].

### Procedures

TKA procedures in this group were performed using prostheses from LINK (Hamburg, Germany). The senior surgeon performed the UKA procedures in this group using the mobile Oxford medial UKA device (Biomet, Bridgend, UK). In the HTO group, we corrected varus malalignment of the lower limb according to Fujisawa's technique, setting the target alignment so that the mechanical axis deviation was 62.5%. The medial end of the tibial plateau was defined as 0%, while the lateral end was defined as 100% [28]. Unless a patient who underwent HTO surgery specifically requests to have the plate removed, our physicians typically do not remove it.

### Statistical analysis

The sample size was estimated using G*Power software (version 3.1.9.7, Düsseldorf, Germany) with ANOVA: fixed effects, omnibus, one-way measures (F test). A power of 0.95 was calculated with an alpha level of 0.05. To detect differences between the three groups, a total sample size of 252 patients was required. In our hospital, over the past five years, the surgeon has performed approximately 150 HTO, 170 UKA, and 300 TKA. The research center possesses a substantial sample size, enabling it to effectively reflect the research findings and better represent the patient characteristics within this region. Consequently, the sample size is comparatively large. Categorical variables were expressed as frequencies or percentages, while continuous data were presented as mean ± standard deviation (SD). The significance of continuous variables was determined using the One-way ANOVA test or Kruskal–Wallis H test, and chi-square tests were used for categorical variables. We conducted a post hoc test using the Mann–Whitney U test with Bonferroni correction. We tested the reliability of FJS-12 using Cronbach’s alpha and analyzed correlations between FJS-12 and other PROMs using Spearman’s correlation coefficients. To predict FJS scores, we used multiple linear regression based on age, BMI, sex, ASA, KL, KS, FS, and surgery (HTO, UKA, and TKA). We added a dummy variable for TKA to use it as a control for HTO and UKA. A *P*-value of < 0.05 was considered statistically significant.

We utilized receiver operating characteristic (ROC) curves to evaluate each PROM's ability to differentiate between patients with and without a forgotten joint. We calculated the area under the curve (AUC) for each ROC. Youden's method was used to determine the forgotten joint MCID for each PROM, with the cutoff point being the maximum Youden index (sensitivity + specificity-1). We conducted all statistical analyses using SPSS v26 (IBM Corporation, Armonk, NY).

## Results

**Fig. 1:**
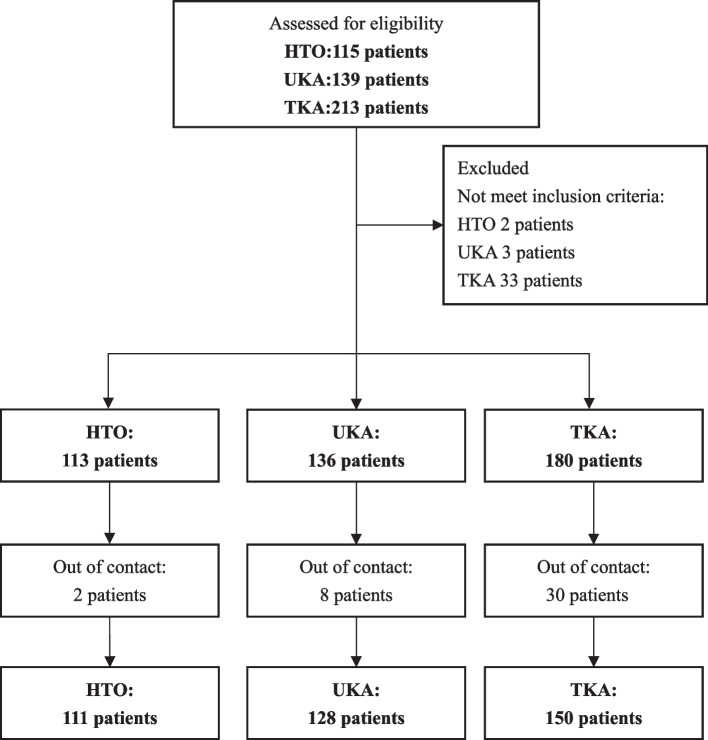
The flow of participants through the study. A total of 467 patients were followed-up after surgery for at least 2 years were included in this retrospective study. In the HTO group, 2 patients who underwent concomitant ACL reconstruction were excluded and 2 patients were lost to follow-up. In the UKA group, 1 patient due to dislocation of the polyethylene insert and 2 patients sustained traumatic injuries were excluded. 8 patients were lost to follow-up. In the TKA group, 1 patient died due to reasons unrelated to the surgery, and 5 patients were excluded due to medical comorbidities. 27 patients were excluded due to the diagnosis of rheumatoid arthritis, and 30 patients were lost to follow-up

Initially, 467 patients underwent a thorough review of their medical records. An additional 38 patients were excluded for not meeting the inclusion criteria, and 40 patients were lost to follow-up. Therefore, the final analysis included 389 patients (Fig. 1).

Table 1 presents the demographic data of the 389 patients. No significant differences were observed in BMI (25.4 ± 3.3,26.3 ± 3.4 and 25.6 ± 3.8, *P* = 0.075), Sex(*P* = 0.773), Side(*P* = 0.930), ASA (*P* = 0.342) and follow up time (46.1 ± 12.9, 48.7 ± 12.6 and 46.1 ± 7.6, *P* = 0.076) among the HTO, UKA, and TKA groups. Patients in the TKA group were significantly older than those in the HTO and UKA groups. Furthermore, significant differences were found between TKA and HTO/UKA groups in terms of mean preoperative KL(*P* < 0.001), ROM (HTO, UKA and TKA:114.5 ± 14.8, 114.3 ± 13.7, 106.7 ± 66.5, *p* < 0.001), KS (HTO, UKA and TKA: 55.0 ± 3.50, 55.5 ± 6.6 and 51.0 ± 4.5, *p* < 0.001), and FS (HTO, UKA and TKA: 63.6 ± 4.3, 63.6 ± 4.4 and 62.5 ± 4.3, *p* < 0.001). Additionally, FS had distinct differences between UKA and TKA groups (63.6 ± 4.4 and 62.5 ± 4.3, *p* = 0.048). Significant differences were found between the TKA group and the other groups in terms of postoperative KS and FS (KS: HTO vs UKA vs TKA = 82.9 ± 7.5 vs 84.2 ± 9.9 vs 80.1 ± 8.2, *p* = 0.001; FS: HTO vs UKA vs TKA = 83.8 ± 8.8 vs 85.9 ± 8.2 vs 79.2 ± 8.5, *p* = 0.001). For the HTO and UKA groups, significant differences were observed in KS (82.9 ± 7.5 and 84.2 ± 9.9, *p* = 0.025), but no significant differences were observed in FS (83.8 ± 8.8 and 85.9 ± 8.2, *p* = 0.061).

**Table 1:** Demographics of participants

	Mean ± SD			
	HTO(*n* = 111)	UKA(*n* = 128)	TKA(*n* = 150)	*P* Value
Age(years)	61.7 ± 5.7	63.25 ± 3.7	67.9 ± 5.9	< 0.001^a^
BMI (kg/m2)	25.4 ± 3.3	26.3 ± 3.4	25.6 ± 3.8	0.075
Sex (%)				0.773
Male	20 (18.0)	19(14.8)	23(15.3)	
Female	91 (82.0)	109(85.2)	127(84.7)	
Side (%)				0.930
Left	51(45.9)	59(46.1)	72(48.0)	
Right	60(54.1)	69(53.9)	78(52.0)	
KL (%)				< 0.001
1	16(14.4)	0(0)	0(0)	
2	59(53.2)	0(0)	0(0)	
3	34(30.6)	88(68.8)	90(0.60)	
4	2(1.8)	40(31.3)	60(0.40)	
ASA (%)				0.342
I	18(16.2)	11(8.6)	24(16.0)	
II	80(72.1)	104(81.3)	110(73.3)	
III	13(11.7)	13(10.2)	16(16.2)	
Follow up(months)	46.1 ± 12.9	48.7 ± 12.6	46.1 ± 7.6	0.076
Preoperative				
ROM				
Flexion(°)	114.5 ± 14.8	114.3 ± 13.7	106.7 ± 66.5	< 0.001^b^
KS	55.0 ± 3.50	55.5 ± 6.6	51.0 ± 4.5	< 0.001^c^
FS	63.6 ± 4.3	63.6 ± 4.4	62.5 ± 4.3	0.027^d^

*SD* Standard deviation, *BMI* Body mass index, *KL* Kellgren–Lawrence, *ASA* American Society of Anesthesiology, *ROM*, Range of motion, *KS* Knee Score, *FS* Function Score

^a^HTO vs UKA:*P* = 0.304. Other pairwise comparisons: *P* < 0.001

^b^HTO vs UKA:*P* = 1.000. Other pairwise comparisons: *P* < 0.001

^c^HTO vs UKA:*P* = 0.694. Other pairwise comparisons: *P* < 0.001

^d^HTO vs UKA:*P* = 1.000. HTO vs TKA: *P* = 0.101. UKA vs TKA: *p* = 0.048

Table 2 compares the HTO, UKA, and TKA groups using FJS in a single variable analysis. A significant difference was found in the total FJS between the HTO, UKA, and TKA groups (59.38 ± 7.25, 66.69 ± 7.44, and 56.90 ± 6.85, respectively; *p* < 0.001). Similarly, a significant difference was found between TKA, UKA, and HTO groups in each of the 12 items of postoperative FJS. However, no significant differences were found in HTO and UKA groups in Q1(0.28 ± 0.54 and 0.39 ± 0.52, *p* = 0.159), Q2(0.97 ± 0.29 and 0.91 ± 0.44, *p* = 0.285), Q3(1.26 ± 0.51 and 1.28 ± 0.60, *p* = 1.000), and Q6(2.18 ± 0.58 and 2.33 ± 0.75, *p* = 0.226). The study revealed that there was no significant difference in joint awareness across various activities, such as lying in bed at night, sitting in a chair, walking for 15 min, and climbing stairs, within both the HTO and UKA groups. These findings indicate that there is little to no variability in joint awareness during daily exercise activities, specifically standing and walking. In the HTO and TKA groups, the questions Q2(0.97 ± 0.29 and 1.03 ± 0.49, *p* = 1.000), Q4(1.16 ± 0.39 and 1.14 ± 0.39, *p* = 1.000), Q8(2.11 ± 0.64 and 2.11 ± 0.61, *p* = 1.000), Q9(2.57 ± 0.64 and 2.59 ± 0.67, *p* = 1.000), and Q10(1.61 ± 0.56 and 1.51 ± 0.55, *p* = 0.416), did not reveal any significant difference in joint awareness during activities such as daily standing. Furthermore, the study found no significant differences in joint awareness among Q1(0.39 ± 0.52 and 0.53 ± 0.60, *p* = 0.192), Q7(1.24 ± 0.45 and 1.87 ± 0.61, *p* = 0.056), and Q12(1.57 ± 0.57 and 2.09 ± 0.54, *p* = 1.000) within the UKA and TKA groups. These results indicate that there were no discernible variations in joint awareness during sleep, walking on uneven surfaces, and performing preferred exercises in both UKA and TKA patients.

**Table 2:** Comparison between HTO, UKA and TKA groups using FJS in a single variable analysis

	Mean ± SD			*P* Value			
	HTO(*n* = 111)	UKA(*n* = 128)	TKA(*n* = 150)	Overall	HTO vs UKA	HTO vs TKA	UKA vs TKA
Total FJS	59.38 ± 7.25	66.69 ± 7.44	56.90 ± 6.85	< 0.001	< 0.001	0.020	< 0.001
Q1	0.28 ± 0.54	0.39 ± 0.52	0.53 ± 0.60	0.001	0.159	< 0.001	0.192
Q2	0.97 ± 0.29	0.91 ± 0.44	1.03 ± 0.49	0.029	0.285	1.000	0.026
Q3	1.26 ± 0.51	1.28 ± 0.60	1.87 ± 0.62	< 0.001	1.000	< 0.001	< 0.001
Q4	1.16 ± 0.39	0.99 ± 0.32	1.14 ± 0.39	0.001	0.002	1.000	0.003
Q5	1.14 ± 0.35	0.95 ± 0.39	1.31 ± 0.49	< 0.001	0.005	0.007	< 0.001
Q6	2.18 ± 0.58	2.33 ± 0.75	2.62 ± 0.66	< 0.001	0.226	< 0.001	0.001
Q7	1.67 ± 0.55	1.24 ± 0.45	1.87 ± 0.61	< 0.001	< 0.001	< 0.001	0.056
Q8	2.11 ± 0.64	1.35 ± 0.54	2.11 ± 0.61	< 0.001	< 0.001	1.000	< 0.001
Q9	2.57 ± 0.64	2.05 ± 0.77	2.59 ± 0.67	< 0.001	< 0.001	1.000	< 0.001
Q10	1.61 ± 0.56	1.15 ± 0.38	1.51 ± 0.55	< 0.001	< 0.001	0.416	< 0.001
Q11	2.64 ± 0.62	1.80 ± 0.58	2.01 ± 0.49	< 0.001	< 0.001	< 0.001	0.015
Q12	2.08 ± 0.54	1.57 ± 0.57	2.09 ± 0.54	< 0.001	< 0.001	< 0.001	1.000

Total FJS is equal to 100 − [(total score for each question)/12 (if there is no response, divided by the number of questions answered) × 25], where the score of each question ‘Are you aware of your artificial joint…,’ from 0 to 4 points (never, 0 points; almost never, 1 point; seldom, 2 points; sometimes, 3 points; mostly, 4 points) *SD*, standard deviation, *FJS*, forgotten joint score-12

Table 3 presents the results of a multiple linear regression analysis predicting the Forgotten Joint Score (FJS) based on age, body mass index (BMI), sex, Kellgren-Lawrence (KL) grade, American Society of Anesthesiologists (ASA) grade, preoperative flexion, Knee Society (KS) score, Functional Score (FS), and type of surgery HTO, UKA, or TKA. The standardized residuals for the model demonstrated a standard normal distribution. The regression analysis resulted in an F value of 18.365 (*P* < 0.001) and an R^2^ value of 0.309, indicating that the model is valid. The predicted FJS can be calculated as 76.644—0.327 (age)—0.116 (BMI)—1.703 (sex) + 0.203 (KL grade) + 0.516 (ASA grade) + 0.848 (if the surgery was HTO) or 8.623 (if the surgery was UKA)—0.01 (preoperative flexion)—0.093 (KS score) + 0.203 (FS). Age is measured in years, BMI in kg/m2, and flexion as an angle. Sex is coded as 1 for male and 2 for female, while KL grade is divided into 1, 2, 3, and 4, and ASA grade is divided into 1, 2, and 3. KS and FS scores are calculated on a hundred-point scale.

**Table 3:** A multiple linear regression calculated to predict FJS based on their age, BMI, sex, KL, ASA, preoperative flexion, KS, FS and received surgery (HTO, UKA and HTO)

	Parameter estimate	Standard error	t value	*p*-value
Intercept	76.644	9.336	8.210	< 0.001
Age (years old)	-0.327	0.073	-4.488	< 0.001
BMI (kg/m^2^)	-0.116	0.101	-1.145	0.253
Sex (male = 1, female = 2)	-1.703	0.971	-1.753	0.080
KL	0.203	0.667	0.304	0.761
ASA	0.516	0.739	0.698	0.486
HTO	0.848	1.263	0.671	0.502
UKA	8.623	1.019	8.460	< 0.001
TKA	0	0	0	0
Preoperative factors				
Flexion(°)	-0.010	0.008	-1.261	0.208
KS	-0.093	0.097	-0.958	0.339
FS	0.203	0.082	2.491	0.013

Participant’s predicted FJS is equal to 76.644- 0.327 (age)-0.116 (BMI) -1.703(sex) + 0.203 (KL grade) + 0.516(ASA grade) + 0.848 (If is HTO) or 8.623(If is UKA) -0.01(Flexion)-0.093(KS) + 0.203(FS)

**Fig. 2:**
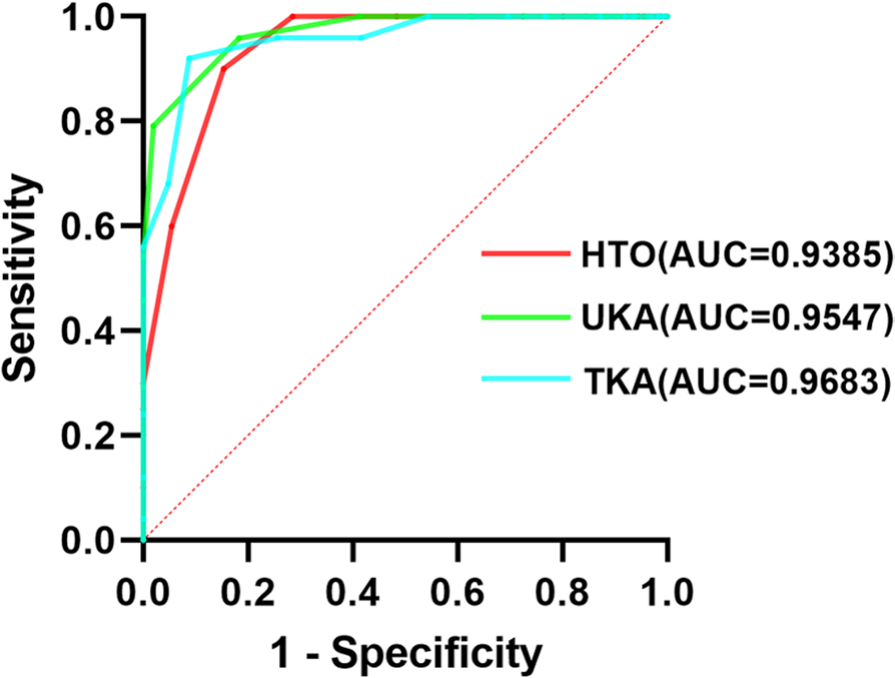
ROC curves of the FJS for the detection of a forgotten joint. ROC, receiver operating characteristic curve; FJS, Forgotten Joint Score-12; **A** high tibial osteotomy (HTO), **B** unicompartmental knee arthroplasty (UKA), **C** total knee arthroplasty (TKA)

Table 4 displays Spearman’s coefficients between clinical outcomes and the anchor (Patient’s Joint Perception [PJP]) questions in three groups. The FJS and anchor (PJP) were strongly correlated (*P* < 0.001). Overall, 20 of the 111 patients (18.0%) in the HTO group, 24 of the 128 patients (18.8%) in the UKA group, and 25 of the 150 patients (16.7%) in the TKA group reported that their joints felt “like a native or natural joint” and were considered to have a forgotten joint. We found that the AUC values in the HTO, UKA and TKA groups were 0.9385, 0.9547 and 0.9683, respectively (Fig. 2). The MCID of the FJS that maximized the sensitivity and specificity for detecting a forgotten joint was 63.54 (sensitivity:0.90, specificity:0.75) in the HTO group, 69.79 (sensitivity:0.958, specificity:0.775) in UKA group and 61.45 (sensitivity:0.92, specificity:0.832) in TKA group. Internal consistency in terms of Cronbach’s alpha was 0.806 for FJS.

**Table 4:** Spearman’s Coefficients Between clinical outcomes and Anchor (PJP) Questions

	HTO		UKA		TKA	
	correlation	*p*-value	correlation	*p*-value	correlation	*p*-value
FJS	-0.826	< 0.001	-0.842	< 0.001	-0.756	< 0.001

*PJP*, Patient’s Joint Perception; *FJS*, forgotten joint score-12

## Discussion

In the present study, we observed a significant difference in the mean FJS among patients who underwent HTO, UKA, and TKA. Our results showed that medial UKA was superior to HTO and TKA in terms of patient awareness. Currently, there is no consensus or studies regarding the outcomes of HTO, UKA, and TKA. Some studies have reported significant differences in FJS between UKA and TKA after an average of one year following surgery, with score values of 73.9 ± 22.8 and 59.3 ± 29.5 for UKA and TKA, respectively (*p* = 0.002) [29]. However, Thienpont et al. found no significant difference in FJS between the UKA and TKA groups after two years following (UKA:76.4 ± 19; TKA: 73.2 ± 22, *p* = 0.436, respectively) [30]. Thus, our finding is similar to the result found by some studies [30–32]. The outcomes of UKA may closely resemble the forgotten knee joint, allowing for greater participation in high-demand activities and resulting in higher satisfaction compared to TKA and HTO. Our speculation is that the observed difference may be attributed to the fact that UKA is a surgical procedure that is more focused on conserving soft tissue and bone compared to TKA. These findings offer valuable insights that can assist surgeons in providing informed guidance to patients who are contemplating HTO, UKA, or TKA.

Although several studies have reported FJS following UKA and TKA [22, 29, 31, 33], there is a paucity of research on FJS after HTO. However, Itoh et al. [18] have demonstrated the reliability and validity of FJS in evaluating patients who have undergone HTO. The FJS-12 questionnaire showed high internal consistency in our study (Cronbach's alpha = 0.806), indicating that it is a reliable tool for evaluating patients who have undergone HTO, UKA, or TKA. A Cronbach's alpha value greater than 0.7 is considered acceptable [34]. Our study revealed a significant difference in FJS between the HTO and UKA groups (Table 3), in contrast to the findings of Watanabe et al. [8] and Jin et al. [35]. The underlying reason for this phenomenon stems from the fact that, while both HTO and UKA aim to preserve soft tissue and bone, the lower FJS scores primarily attributed to alterations in the lower limb force line induced by HTO. The other reason could be attributed to plate irritation of the skin and subcutaneous tissue of the proximal medial lower leg, which may reduce postoperative subjective satisfaction, particularly in younger patients with high functional activity requirements. Another possible explanation is that the plate used in the HTO patients in the studies by Watanabe et al. [8] and Jin [35] was removed during the last follow-up. Some reports have shown that HTO is superior to UKA in ROM [36, 37], and no certainty has been reached. In our results, UKA was inferior postoperative ROM compared to those of HTO (Table 2), which is consistent with the above study.

In our study, we found that a female at, younger age leads to lower FJS (Table 4). Discrepancies in baseline scores have the potential to confound the assessment of PROMs [38]. Younger patient age has been identified as a negative predictor of favorable postoperative PROMs [39]. Additionally, younger patients tend to exhibit higher preoperative expectations for resuming high-demand activities compared to their older counterparts [40]. Both activity levels and expectations of activities gradually decline with increasing age [39, 40]. Age has emerged as the most influential factor among the preoperative patient characteristics in relation to postoperative PROMs. Among these characteristics, age showed the strongest association with postoperative PROMs. Our results were aligned with the results of Li et al. [41], who reported that females, younger age, and higher BMI have lower FJS in patients before TKA. Our results confirm that preoperative FS would predict better FJS.

The FJS MCID for HTO, UKA, and TKA were 63.54, 69.79, and 61.45, respectively. As there are no prior studies reporting FJS MCID for these procedures, comparisons cannot be made. However, Wang et al. [17] measured FJS MCID for a forgotten joint in UKA patients and found that an FJS of > 84.38 corresponds to a forgotten joint when using the same anchor question. The reasons for the discrepancy are unclear, and further investigation is necessary. These MCID may be useful for surgeons to interpret FJS scores in daily practice. Scores above the MCID suggest that the patient has achieved a forgotten joint status, which is defined by the anchor question as feeling like they have a natural joint. The anchor question used in this study reflects the patients’ joint perception, with the best answer being “like a native or natural joint,” which is consistent with the concept of a forgotten joint [17]. Our study found that 18.0% of HTO, 18.75% of UKA, and 16.67% of TKA patients achieved a forgotten joint. Furthermore, older patients undergoing TKA surgery achieved a forgotten joint more frequently than those undergoing HTO and UKA surgery, with TKA MCID being lower than those in the other two groups. This finding may be explained by the fact that younger patients, particularly those undergoing HTO and UKA, have higher activity levels and expectations than older patients and may be more sensitive to joint perception during daily activities [27].

However, there are several limitations to this study. First, evaluating patient joint awareness was challenging due to the subjective nature of the outcome. Although the scoring systems and questionnaire used in this study have been validated, are widely utilized, and self-administered, the questions could not comprehensively capture all aspects of patient satisfaction. Second, typical dichotomous anchor questions [42] were not used in our study. However, the cutoff was clear and reliable [27]. For the forgotten joint, only the answer "like a native or natural joint" was selected because the second answer "like an artificial joint with no restriction" shows that the patient has an artificial joint. Third, the extensive number of follow-up time points increases the potential risk of type 1 statistical error and the risk of selection bias in this retrospective cohort. Four, the correlation between an individual question in the FJS score and clinical practice exhibits bias and unreliability, potentially introducing errors in clinical interpretation. Consequently, this study investigates the assessment of joint awareness using the comprehensive FJS score encompassing all three joints Finally, all of our study subjects were Chinese, and ethnic and cultural differences in this population may affect the FJS MCID. Additionally, this was a predominantly female cohort, which may limit the generalizability of the findings. In the future, more prospective cohorts with rigorous criteria are needed to confirm our results in other patient populations.

## Conclusion

Our study suggests that medial UKA yields lower patients’ awareness than HTO and TKA. To enhance the overall outcome of patients who undergo knee arthroplasty, this study proposes the pursuit of joint-conserving surgical strategies whenever feasible. These findings may provide helpful information for surgeons counseling patients considering HTO、UKA or TKA. Additionally, younger age and higher FS were identified as significant predictors of better FJS through multiple linear regression analysis. The FJS MCID for HTO, UKA, and TKA were found to be 63.54, 69.79, and 61.45, respectively. These MCID can assist surgeons in interpreting FJS scores during clinical follow-up and provide practical reference values for clinical operation options.

## Data Availability

The authors declare that all the data supporting the fndings of this study are available within the article.
